# Pleiotropic effects of statins in distal human pulmonary artery smooth muscle cells

**DOI:** 10.1186/1465-9921-12-137

**Published:** 2011-10-14

**Authors:** Omar F Ali, Ellena J Growcott, Ghazwan S Butrous, John Wharton

**Affiliations:** 1 Centre for Pharmacology and Therapeutics, Imperial College London, Hammersmith Hospital, Du Cane Road, London W12 ONN, UK; 2 Novartis Institute for Biomedical Research, Wimblehurst Road, Horsham, West Sussex RH12 5AB, UK; 3 Division of Cardiopulmonary Sciences, University of Kent, Research and Development Centre, Kent Institute of Medicine and Health Sciences, Parkwood Rd, Canterbury, Kent CT2 7PD, UK

## Abstract

**Background:**

Recent clinical data suggest statins have transient but significant effects in patients with pulmonary arterial hypertension. In this study we explored the molecular effects of statins on distal human pulmonary artery smooth muscle cells (PASMCs) and their relevance to proliferation and apoptosis in pulmonary arterial hypertension.

**Methods:**

Primary distal human PASMCs from patients and controls were treated with lipophilic (simvastatin, atorvastatin, mevastatin and fluvastatin), lipophobic (pravastatin) and nitric-oxide releasing statins and studied in terms of their DNA synthesis, proliferation, apoptosis, matrix metalloproteinase-9 and endothelin-1 release.

**Results:**

Treatment of human PASMCs with selected statins inhibited DNA synthesis, proliferation and matrix metalloproteinase-9 production in a concentration-dependent manner. Statins differed in their effectiveness, the rank order of anti-mitogenic potency being simvastatin > atorvastatin > > pravastatin. Nevertheless, a novel nitric oxide-releasing derivative of pravastatin (NCX 6550) was effective. Lipophilic statins, such as simvastatin, also enhanced the anti-proliferative effects of iloprost and sildenafil, promoted apoptosis and inhibited the release of the mitogen and survival factor endothelin-1. These effects were reversed by mevalonate and the isoprenoid intermediate geranylgeranylpyrophosphate and were mimicked by inhibitors of the Rho and Rho-kinase.

**Conclusions:**

Lipophilic statins exert direct effects on distal human PASMCs and are likely to involve inhibition of Rho GTPase signalling. These findings compliment some of the recently documented effects in patients with pulmonary arterial hypertension.

## Background

It is recognised that 3-hydroxy-3-methylglutaryl-coenzyme A (HMG-CoA) reductase inhibitors (statins) have beneficial cardiovascular effects beyond cholesterol lowering [1,2]. These so-called pleiotropic effects depend principally on inhibiting the synthesis of the isoprenoid intermediates farnesylpyrophosphate (FPP) and geranylgeranylpyrophosphate (GGPP), which are essential for the post-translational processing, membrane translocation and activation of the Ras and Rho GTP-binding protein families. These GTPases regulate many cellular functions and couple membrane growth factor receptors to intracellular pathways that affect cell proliferation [3,4]. Activation of RhoA and its downstream mediator Rho-associated kinase is implicated in the pathogenesis of pulmonary hypertension (PH) and inhibition of the RhoA/Rho-kinase may also contribute to the beneficial effects of established therapies, such as sildenafil [5-8]. Statins inhibit RhoA/Rho-kinase signalling by suppressing mevalonate and GGPP synthesis and have been shown to attenuate the development of PH in several animal models [9-16]. More importantly, simvastatin reversed established experimental pulmonary hypertension [17,18] and this was associated with increased apoptosis and reduced proliferation of smooth muscle cells in vascular lesions [9,17]. The addition of simvastatin to sildenafil also reversed hypoxia-induced pulmonary hypertension and remodelling [16]. In keeping with findings in animal experiments, recent clinical study using simvastatin in PAH showed transient but significant effects on right ventricular mass and NT-proBNP [19].

Differences have emerged in the protective effect of HMG-CoA reductase inhibitors in experimental models of PH [20,21], raising questions about whether statins as a class of drugs are capable of inducing similar responses in the pulmonary vasculature of humans and laboratory animals. Actually, the potential direct effects of statins on the growth and survival of PASMCs are unclear and cells from different regions of the pulmonary vascular bed may vary in their response [22].

We hypothesised that statins have the potential to directly affect proliferation and apoptosis of distal human PASMCs. Specifically, we sought to establish (1) the effect of statins on PASMC proliferation, apoptosis and production of factors (endothelin-1 and matrix metalloproteinase-9) implicated in the pathogenesis of PAH; (2) the anti-proliferative effect of statins when used in combination with established therapies for PAH and (3) the intermediates in the mevalonate pathway responsible for the action of statins.

## Methods

### Cell isolation and culture

PASMCs were derived from micro-dissected segments of distal pulmonary arteries (< 1 mm external diameter) [23,24]. Lung tissues were obtained from patients (8 female/8 male; aged 49.9 ± 2.8 years) undergoing lobectomy or pneumonectomy for bronchial carcinoma (*n *= 3), lung transplantation for idiopathic PAH (IPAH, *n *= 3), emphysema or fibrotic lung disease (*n *= 9) and from unused donor lungs (*n *= 1). Informed consent and approval from the Hammersmith Hospitals (Ref. No. 2001/6003) and Royal Brompton & Harefield Hospitals (Ref. No. 01-210) ethics committees was obtained. Cells (passages 3 to 12) were phenotyped using immunohistochemical and receptor binding techniques and, like smooth muscle cells in the medial layer of intact distal human pulmonary arteries, they expressed α-smooth muscle actin, calponin, endothelin ET_A _and ET_B _receptors and phosphodiesterase type 5 [23,24]. Cells were quiesced for at least 24 hours in serum-free Dulbeco's modified Eagle medium (DMEM) prior to treatment with statins and other drugs at stated concentrations.

Simvastatin was acquired both as a pro-drug (activated by alkaline hydrolysis) and in active form, whereas other statins were obtained as active compounds (Merck Biosciences Ltd., Nottingham, UK). Nitric oxide (NO)-releasing derivatives of pravastatin (NCX 6550) and fluvastatin (NCX 6553) were provided by the NiCox Research Institute, Milan, Italy [25]. Involvement of specific signalling pathways was assessed by examining the ability of exogenous mevalonic acid (MVA), squalene, geranylgeranylpyrophosphate (GGPP) and farnesylpyrophosphate (FPP) to reverse responses to statin treatment and using inhibitors of geranylgeranyl transferase (GGTI-2133), farnesyl transferase (FTI-277), Rho (exoenzyme C3) and Rho-kinase (Y-27632) (Merck Biosciences Ltd.).

### DNA synthesis

DNA synthesis was assessed by measuring incorporation of [^3^H-methyl]-thymidine (0.25 μCi/well; GE Healthcare, Little Chalfont, Buck's, UK) over 24 hours in cells stimulated with recombinant human platelet-derived growth factor (PDGF, 5 ng/ml; R&D Systems Europe Ltd., Abingdon, Oxon, UK) [24,26]. Some experiments were conducted using statins in combination with iloprost (GE Healthcare) and sildenafil (Pfizer Global Research & Development, Sandwich, Kent, UK).

### Cell viability and proliferation

Cell viability was assessed by measuring trypan blue exclusion and ATP content using a CellTiter-Glo^® ^Luminescent cell viability assay (Promega Coporation, Southampton, UK). Adherent cells were trypsinized and counted with a multi-chamber haemocytometer.

### Apoptosis

Apoptosis was assessed by measuring cytoplasmic histone-associated DNA fragments (Roche Diagnostics Ltd, Lewis, Sussex, UK) and Hoechst 33342 staining [24,26], in the presence and absence of the pan-caspase inhibitor z-VAD-fmk (MP Biomedicals Europe, Illkirch, France).

### Endothelin-1 and matrix metalloproteinase-9 production

Production of ET-1 was stimulated with recombinant human transforming growth factor-β1 (TGF-β1, 10 ng/ml; R&D Systems) for 24 hours and matrix metalloproteinase-9 (MMP-9) by tumour necrosis factor-α (TNF-α, 10 ng/ml) and phorbol 12-myrisate 13-acetate (PMA, 0.1 μM). ET-1 and MMP-9 were measured in conditioned medium using QuantiGlo^® ^(R&D Systems, UK) and Biotrack^® ^immunoassays (GE Healthcare, UK) respectively [24].

### Statistical analysis

Data are expressed as means ± SEM and analysed with GraphPad Prism version 4.0 (GraphPad Software, San Diego, CA). Comparisons were made by one-way ANOVA with Tukey's post test and Student's *t *test as appropriate. A probability of *P *< 0.05 indicated statistical significance.

## Results

### Statin treatment reduces cell proliferation and promotes apoptosis

**Figure 1:**
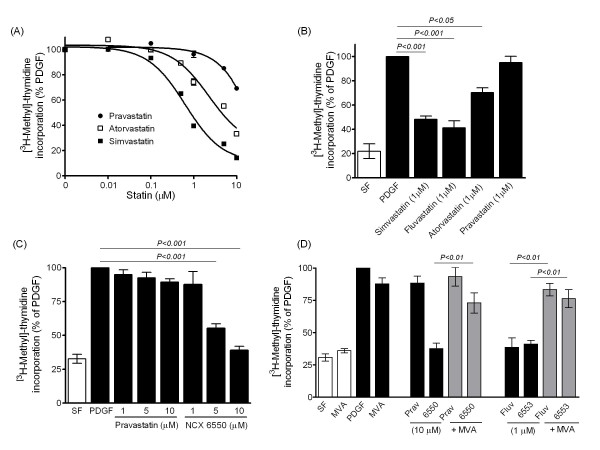
**Effect of statins on DNA synthesis in human PASMCs**. (A-B) Inhibition of DNA synthesis, assessed by PDGF-stimulated (5 ng/ml) [^3^H-methyl]-thymidine incorporation, by statins with rank-order of potency simvastatin = fluvastatin > atorvastatin > > pravastatin. (C-D) Effect of NO-releasing derivative of pravastatin (NCX 6550), fluvastatin and NO-releasing derivative of fluvastatin (NCX 6553) and prevention by mevalonate (MVA, 100 μM). MVA alone had no significant effect on DNA synthesis in SF and PDGF-stimulated cells. Data are mean ± SEM from 3-4 distinct PASMC isolates (A-B, D) or replicate values (*n *= 6) representative of independent experiments (C), using IPAH (B, D), and non-IPAH (A, C) cells. SF = serum-free untreated controls.

Treatment of human PASMCs with PDGF increased DNA synthesis ~4-fold (*P *< 0.001) and was attenuated by statins in a concentration-dependent manner. This effect was most marked when concentrations above 0.1 μM were used, with 1 μM resulting in significant inhibition in both IPAH and non-IPAH cells (Figure 1A-B). However, statins differed in their relative potency, with a rank order of simvastatin (IC_50 _0.68 ± 0.09 μM, *n *= 4 isolates) > atorvastatin (IC_50 _3.11 ± 0.84 μM, *n *= 3 isolates; *P *= 0.019) > > pravastatin (IC_50 _> 100 μM, *n *= 3 isolates) (Figure 1A). While pravastatin (1-10 μM) failed to significantly inhibit DNA synthesis the NO-releasing derivative NCX 6500 was effective at concentrations of 5-10 μM (5-fold higher concentration as compared to other lipophilic statins) (Figure 1C). Fluvastatin and its NO-releasing derivative NCX 6553 (1 μM ) inhibited DNA synthesis to a similar extent and the effects of all three compounds were reversed by MVA (Figure 1D).

**Figure 2:**
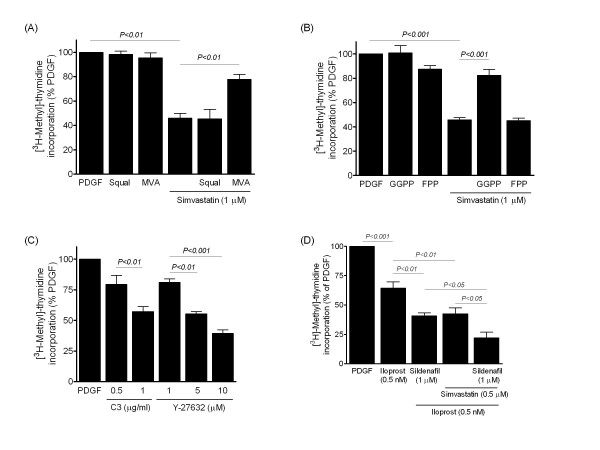
**Effects of modulating the mevalonate pathway and combination therapy on DNA synthesis in human PASMCs**. (A-B) Effect of simvastatin and reversal by the addition of mevalonate (MVA, 100 μM) or geranylgeranylpyrophosphate (GGPP, 10 μM), but not by squalene (Squal, 100 μM) or farnesylpyrophosphate (FPP, 10 μM). (C) Inhibitory effect of exoenzyme C3 (C3) and Rho-kinase inhibitor Y-27632. (D) Inhibitory effect of iloprost in combination with sildenafil or simvastatin and co-treatment with all three agents. Data are mean ± SEM of replicate values (*n *= 6), representative of independent experiments, and were obtained using IPAH (D) and non-IPAH (A-C) cells.

**Figure 3:**
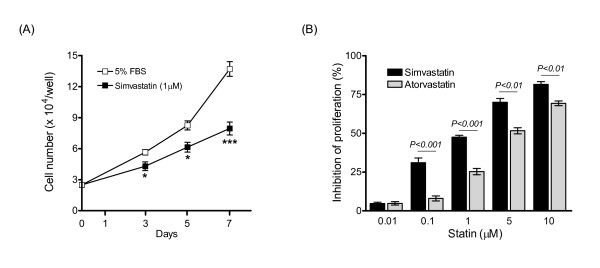
**Effect of statins on cell counts**. (A-B) Inhibition of serum-stimulated (5% FBS) proliferation; simvastatin being more potent than atorvastatin at equimolar concentrations. Data are mean ± SEM of replicate values, representative of independent experiments with IPAH cells (A), and of distinct non-IPAH PASMC isolates (*n *= 4) (B). *, *P *< 0.05; ***, *P *< 0.001 versus control cells.

The inhibitory effect of simvastatin was reversed by MVA and GGPP, but not by the cholesterol precursor squalene or by FPP (Figure 2A-B); indicating that the anti-proliferative effect was due to inhibition of HMG-CoA reductase and isoprenylation of Rho proteins and not to the interruption of cholesterol synthesis. Inhibitors of Rho and Rho-kinase also attenuated DNA synthesis, implicating Rho/Rho-kinase signalling in the mitogenic response to PDGF (Figure 2C). In addition, the anti-proliferative effect of the prostacyclin analogue iloprost and PDE5 inhibitor sildenafil on human PASMCs [22,26] was enhanced when used together with simvastatin, the combined effect being greater than either agent alone (Figure 2D). Serum-stimulated proliferation of PASMCs from patients with IPAH was also attenuated by statins, simvastatin being more potent than atorvastatin at equimolar concentrations (Figure 3A-B). The proportion of adherent, non-viable trypan blue-stained PASMCs was relatively low (1-2%) and no significant acute toxic effects were observed with increasing statin concentrations, as determined by assessing intracellular ATP levels over 24 hours in both the presence and absence of serum (data not shown).

**Figure 4:**
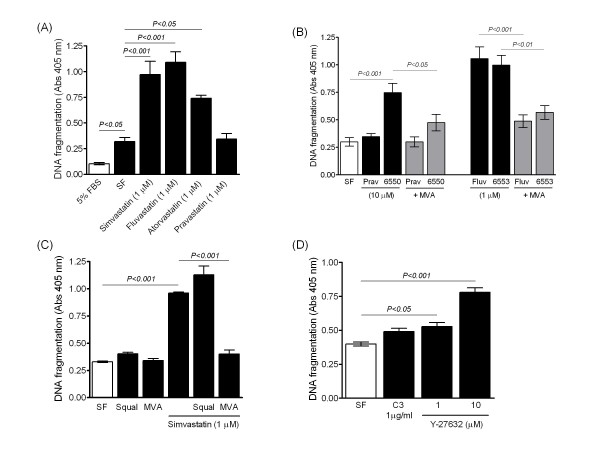
**Pro-apoptotic effect of statins, as assessed by DNA fragmentation**. (A) Serum deprivation (SF) increased DNA fragmentation, compared to PASMCs cultured in presence of serum (5% FBS). Lipophilic statins such as simvastatin, fluvastatin and atorvastatin, but not the lipophobic pravastatin, promoted apoptosis in serum deprived cells. (B-C) Pro-apoptotic effect of simvastatin, fluvastatin and NO-releasing derivatives of pravastatin (6550) and fluvastatin (6553) reversed by mevalonate (MVA, 100 μM), but not by squalene (Squal, 100 μM). (D) Pro-apoptotic effect of exoenzyme C3 (C3) and Rho-kinase inhibitor Y-27632. Data are mean ± SEM of replicate values (*n *= 4) and representative of independent experiments with IPAH (A-B) and non-IPAH (C-D) cells. SF = serum free untreated cells.

**Figure 5:**
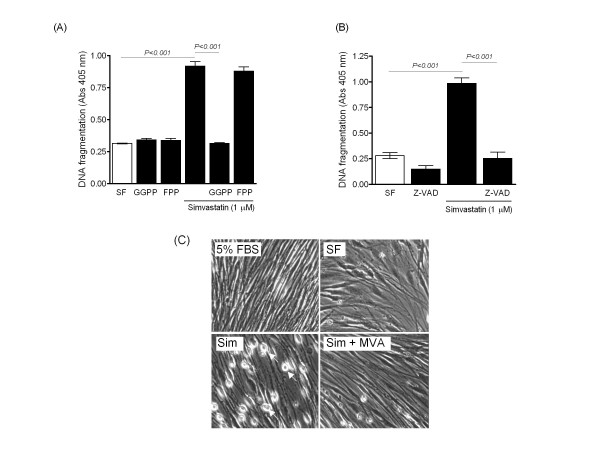
**Effect of simvastatin on DNA fragmentation and PASMC morphology**. (A-B) Pro-apoptotic effect of simvastatin reversed by geranylgeranylpyrophosphate (GGPP, 10 μM) and the pan-caspase inhibitor Z-VAD-fmk (Z-VAD, 10 μM), but not by farnesylpyrophosphate (FPP, 10 μM). (C) PASMCs showed characteristic morphological changes (arrows) following simvastatin-treatment (Sim, 1 μM), which were prevented by co-administration of mevalonate (MVA, 100 μM). Data are mean ± SEM of replicate values (*n *= 4) and representative of independent experiments with non-IPAH (A) and IPAH cell isolates (B). SF = serum free untreated cells.

Serum-deprivation increased DNA fragmentation and this was augmented by simvastatin, fluvastatin and NO-releasing derivatives of pravastatin (NCX 6500) and fluvastatin (NCX 6553), but not by pravastatin (Figure 4A-B). Theses pro-apoptotic effects were reversed by MVA, but not by squalene, and mimicked in cells treated with Y-27632 (Figure 4C-D). The effect of statins was also prevented by GGPP, but not FPP, and abolished by the pan-caspase inhibitor z-VAD-fmk (Figure 5A-B). Furthermore, the pro-apoptotic effect was verified by assessment of nuclear chromatin condensation in Hoechst-stained cells (Additional File 1) and accompanied by morphological changes. Cultured cells typically became rounded and isolated from their neighbours following statin treatment and this was prevented by MVA (Figure 5C).

### Statin treatment inhibits ET-1 release and MMP-9 production

**Figure 6:**
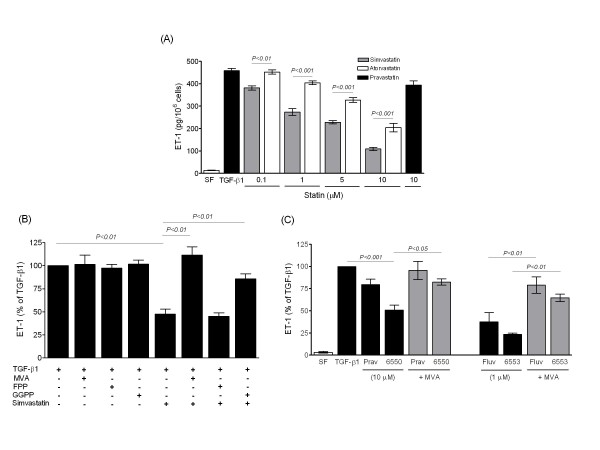
**Effect of statins on TGF-β1-stimulated (10 ng/ml) ET-1 release from human PASMCs**. (A) Release of ET-1 in conditioned medium was attenuated in a concentration-dependent manner by lipophilic statins, but not by pravastatin, with simvastatin being more potent than atorvastatin. (B) Effect of simvastatin reversed by co-treatment with mevalonate (MVA, 100 μM) or geranylgeranylpyrophosphate (GGPP; 10 μM), but not by farnesylpyrophosphate (FPP; 10 μM). (C) Inhibitory effect of fluvastatin and NO-releasing derivatives of pravastatin (6550) and fluvastatin (6553) also reversed by MVA. Data are mean ± SEM of replicate values (*n *= 4) from representative IPAH cell isolates and expressed either as the amount of ET-1 detected (per 10^6 ^cells) in conditioned medium (A) or as a proportion of that measured in control cells treated with TGF-β1 alone (B-C). SF = serum free untreated cells.

**Figure 7:**
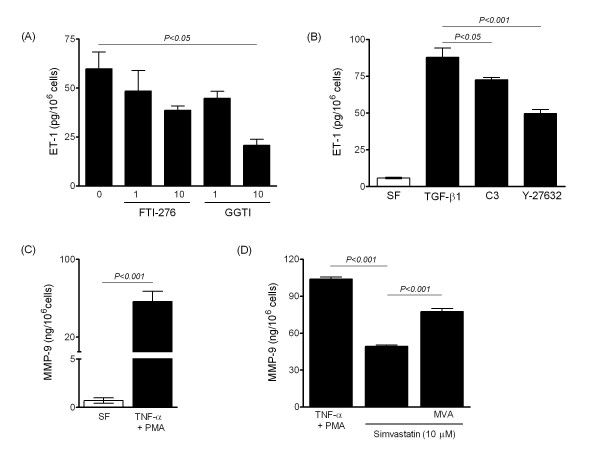
**Modulation of ET-1 and matrix metalloproteinase-9 (MMP-9) production in human PASMCs**. (A) Inhibition of geranylgeranyl transferase (GGTI-2133), rather than farnesyl transferase (FTI-277) mimicked the statin affect, inhibiting ET-1 release. (B) Inhibitory effect of exoenzyme C3 (C3, 1 μg/ml) and the Rho kinase inhibitor Y-27632 (10 μM) on ET-1 release. (C) Induction of MMP-9 production following dual stimulation with TNF-α (10 ng/ml) and PMA (0.1 μM) in serum-free (SF) medium. (D) Inhibition of MMP-9 production by simvastatin and reversal by mevalonate (MVA, 100 μM). Data are mean ± SEM of replicate values that are representative of independent experiments with non-IPAH cells (A, B, D) and 5-6 distinct PASMC isolates (C).

Human PASMCs represent an important site of ET-1 production, particularly when stimulated with cytokines or growth factors such as TGF-β1 [23,27]. Lipophilic statins inhibited ET-1 release in a concentration-dependent manner from PASMCs isolated from patients with IPAH (Figure 6A), and these inhibitory effects were reversed by the addition of MVA or GGPP, but not FPP (Figure 6B). Pravastatin was again found to be ineffective, whereas the NO-releasing derivative of pravastatin (NCX 6500), fluvastatin and NCX 6553 all attenuated ET-1 production (Figure 6C). Inhibition of farnesyl transferase resulted in non-significant reduction of ET-1 production. Instead inhibition of geranylgeranyl transferase mimicked the effect of statins (Figure 7A) and, consistent with signalling via geranylgeranylated proteins, inhibitors of Rho and Rho-kinase also attenuated ET-1 production (Figure 7B). Stimulation of PASMCs with TNF-α and PMA markedly induces MMP-9 expression and increases MMP-9 activity in conditioned medium [24]. Simvastatin attenuated MMP-9 production from PASMCs which was reversed by MVA (Figure 7C-D).

As might be expected, the magnitude of statin-induced responses varied between different human PASMC isolates. Nonetheless, statins appeared to exhibit reproducible effects in cells from patients with IPAH and those with other lung diseases or apparently normal lung tissues.

## Discussion

We have shown that statins exhibit several complementary effects in distal human PASMCs derived from patients with IPAH as well as other lung diseases. Specifically, lipophilic statins used at concentrations ≥ 1 μM attenuated proliferation, promoted apoptosis and inhibited production of ET-1 and MMP-9, all of which are implicated in the pathogenesis of PAH and remodelling of pulmonary arteries. When used in combination with established therapies for PAH, simvastatin (0.5 μM) also exhibited an additional inhibitory effect on DNA synthesis.

The anti-proliferative effect of statins was dependent on inhibition of the mevalonate pathway and formation of isoprenoids and was selectively reversed by GGPP and not FPP, suggesting that post-translational geranylgeranylation of proteins contributes to the mitogenic effect of PDGF in PASMCs. The Rho family GTPases are in fact a major target of geranylgeranylation and the inhibition of Rho and Rho-kinase is considered to underlie many of the pleiotropic effects of statins in smooth muscle cells [3]. The anti-mitogenic effect of Rho and Rho-kinase inhibitors in PASMCs supports this view. Moreover, activation of RhoA/Rho-kinase signalling is implicated in the pathogenesis of PH and inhibition of this pathway attenuates the development of the disease in experimental models [5,6,16].

Similar effects of statins in other smooth cells have been previously reported. The inhibitory effect of simvastatin was mediated by geranylgeranylation of RhoA but not farnesylation of Ras in bronchial smooth muscle cells [28]. Mevalonate also reversed anti-proliferative effect of simvastatin in human bronchial smooth muscle cells [29]. In addition to the mevalonate pathway, Insulin-like growth factor I/insulin dependent pathway has been implicated in the inhibition of human vascular smooth muscle cells proliferation by lovastatin [30].

Established therapies, such as iloprost and sildenafil display cAMP- and cGMP-dependent anti-proliferative effects in human PASMCs [22,26] and when used together with a statin had a greater inhibitory effect than either agent alone. The use of adjunctive simvastatin in patients with PAH resulted in short-term reduction of right ventricular mass and marker of cardiac failure supporting the concept that combination therapies may be of some benefit, although this has not yet been shown to improve functional capacity [19,31]. Both cAMP and cGMP pathways regulate the activity and expression of RhoA in vascular smooth muscle cells [32] and the beneficial effects of sildenafil and simvastatin in hypoxia-induced PH depend, at least in part, on the inhibition of RhoA- and Rho-kinase-dependent functions [8,18].

A number of apoptosis-based strategies have been successful in reversing pulmonary vascular remodelling in animals [33,34], including the use of simvastatin in monocrotaline- and hypoxia-induced PH [17,18]. However, the apoptotic response to statins varies between different species and cell types [35] and here we establish that statins have a pro-apoptotic effect on isolated distal human PASMCs. The growth of these cultured cells depends on the autocrine production of ET-1 [24], which is a recognized survival factor as well as mitogen and protects against apoptosis in vascular smooth muscle cells [36]. Human PASMCs synthesise substantial amounts of ET-1 when stimulated with TGF-β1 [24] and lipophilic statins such as simvastatin attenuated ET-1 release. In accordance with the Rho-dependent regulation of endothelial ET-1 expression by statins [37], the effect was also mimicked by inhibition of geranylgeranyl transferase, Rho and Rho-kinase. Thus, a reduction in ET-1 release may contribute to both the anti-proliferative and pro-apoptotic effects of statins in PASMCs. Proteolytic enzymes are also implicated in the migration, proliferation and resistance to apoptosis of PASMCs [38,39] and represent another potential therapeutic target as PASMCs exhibit increased gelatinase activity in PAH [40,41] and statins such as simvastatin attenuated MMP-9 production in cultured cells. The precise mechanisms underlying this effect are not fully understood, but include inhibition of RhoA/Rho-kinase activation and reduced MMP-9 mRNA expression [41].

When used at equimolar concentrations, statins differed in their effectiveness (simvastatin > atorvastatin >> pravastatin) at inhibiting DNA synthesis, proliferation and ET-1 production. The ability of statins to inhibit HMG-CoA reductase activity in other non-hepatic human cells is considered to vary with their lipophilicity, simvastatin being 5- to 40-fold more potent than atorvastatin and ~600-fold more potent than pravastatin [42]. Conversely, these statins were broadly equipotent at inhibiting enzyme activity in a cell-free system [42]. Pravastatin had little or no effect on PASMCs unless it was linked to a NO-releasing moiety. Besides slowly releasing NO, this functional group is thought to increase lipophilicity and aid penetration into cells, thereby contributing to the MVA-dependent effects of the NO-releasing derivative NCX 6550 [25]. Thus, unlike pravastatin, lipophilic fluvastatin and its NO-releasing derivative NCX 6553 displayed comparable inhibitory effects on PASMCs. These novel agents have not been studied before in distal human PASMCs and could be used as tools in interrogating deranged cellular pathways in PAH.

It is worth noting that the response of IPAH and non-IPAH cells to lipophilic statins may be different, although such a difference was not apparent when statins were used at concentrations ≥ 1 μM in our studies. A recent study suggested differences in PDGF (10 ng/ml) induced cell proliferation between IPAH and non-IPAH cells when treated with 0.1 μM of simvastatin [43]. The authors demonstrated greater inhibitory effect of IPAH than non-IPAH cells. However, in the same study 1 μM of simvastatin exhibited significant inhibitory effect in non-IPAH cells consistent with our findings. Thus medium to high concentrations of lipophilic statins affect non-IPAH and IPAH cells equally. Although the doses of statins used in our experiments are similar to other studies, such concentrations are unlikely to be achieved in human plasma. It is conceivable that lipophilic statins used in clinical doses could result in a cumulative response through their effects on a number of cell systems. Speculatively our *in vitro *observations could explain the short-term effects of simvastatin in the recent clinical study in PAH [19].

## Conclusions

Lipophilic statins directly modulate proliferation, apoptosis and the production of ET-1 and MMP-9 in human PASMCs. These effects are relevant to the pathogenesis of PAH.

## List of Abbreviations

ET-1: endothelin-1; FPP: farnesylpyrophosphate; GGPP: geranylgeranylpyrophosphate; HMG-CoA reductase: 3-hydroxy-3-methylglutaryl-coenzyme A reductase; IPAH: idiopathic pulmonary arterial hypertension; MMP-9: matrix metalloproteinase-9; MVA: mevalonate; NO: nitric oxide; PAH: pulmonary arterial hypertension; PASMCs: pulmonary artery smooth muscle cells; PDGF: human platelet-derived growth factor; PH: pulmonary hypertension; PMA: phorbol 12-myrisate 13-acetate; TGF-β1: transforming growth factor-β1; TNF-α: tumour necrosis factor-α.

## Competing interests

The authors declare that they have no competing interests.

## Authors' contributions

OA carried out all the cell-based assays. EG participated in parts of the ET-1/MMP-9 experiments design and analysis. GB helped in the production of the manuscript. JW supervised the entire project and helped in the data analysis and manuscript preparation. All authors have read and approved the final manuscript.

## Supplementary Material

Additional File 1 **Pro-apoptotic effect of simvastatin, as assessed by Hoechst staining**. (A) Simvastatin-treated PASMCs show characteristic nuclear condensation (arrows). (B-C) Pro-apoptotic effect of simvastatin in serum-deprived cells reversed by mevalonate (MVA, 100 μM), geranylgeranylpyrophosphate (GGPP; 10 μM) and the pan-caspase inhibitor Z-VAD-fmk (50 μM). Data are mean ± SEM from 3-4 distinct cell isolates. SF = serum-free untreated cells. Click here for file
