# Supplementary figures and images for: Pleiotropic effects of statins in distal human pulmonary artery smooth muscle cells
Source: Respir Res. 2011 Oct 14;12(1):137. doi: 10.1186/1465-9921-12-137 (PMC3213146; doi:10.1186/1465-9921-12-137)

## Slide 1
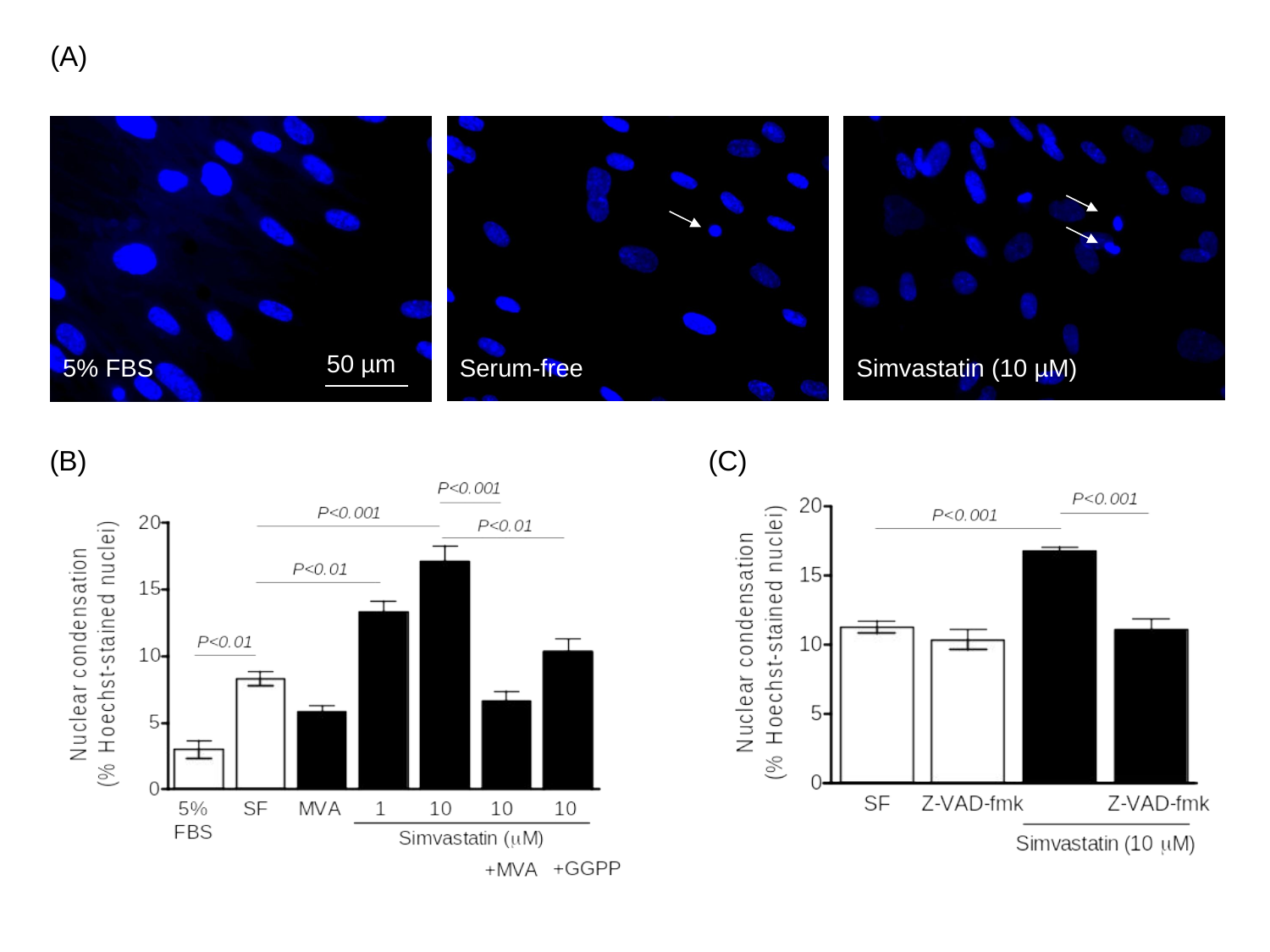

(A)
 50 µm
5% FBS
Serum-free
Simvastatin (10 µM)
(B)
(C)

Supplement: Additional File 1 — Pro-apoptotic effect of simvastatin, as assessed by Hoechst staining. (A) Simvastatin-treated PASMCs show characteristic nuclear condensation (arrows). (B-C) Pro-apoptotic effect of simvastatin in serum-deprived cells reversed by mevalonate (MVA, 100 μM), geranylgeranylpyrophosphate (GGPP; 10 μM) and the pan-caspase inhibitor Z-VAD-fmk (50 μM). Data are mean ± SEM from 3-4 distinct cell isolates. SF = serum-free untreated cells. [file 1465-9921-12-137-S1.PPT]
